# Experimental estimation and analysis of variance of the measured loss power of magnetic nanoparticles

**DOI:** 10.1038/s41598-017-07088-w

**Published:** 2017-07-27

**Authors:** Frederik Soetaert, Sri Kamal Kandala, Andris Bakuzis, Robert Ivkov

**Affiliations:** 1 Department of Electrical Energy, Systems and Automation, Ghent University, Technology park 913, B-9052 Zwijnaarde, Belgium; 2 Department of Radiation Oncology and Molecular Radiation Sciences, The Johns Hopkins University School of Medicine, Baltimore, MD 21231 USA; 3 Department of Mechanical Engineering, Whiting School of Engineering, The Johns Hopkins University, Baltimore, MD 21218 USA; 4 Instituto de Física, Universidade Federal de Goiás, 74690-900 Goiânia-GO, Brazil; 5 Department of Oncology, The Johns Hopkins University School of Medicine, Baltimore, MD 21231 USA; 6 Institute for NanoBioTechnology, The Johns Hopkins University, Baltimore, MD 21218 USA; 7 Department of Materials Science and Engineering, Whiting School of Engineering, The Johns Hopkins University, Baltimore, MD 21218 USA

## Abstract

Magnetic nanoparticles dissipate heat when exposed to alternating magnetic fields (AMFs), making them suitable for cancer hyperthermia. Therapeutic heating applications demand accurate characterization of the heating power dissipated by the particles. Specific loss power (SLP) generated by magnetic nanoparticles is estimated from calorimetric heating measurements. Such measurements require adiabatic conditions, yet they are typically performed in an AMF device with non-adiabatic conditions. We have measured heating from four magnetic nanoparticle constructs using a range of frequencies (150–375 kHz) and magnetic fields (4–44 kA/m). We have extended a method developed to estimate SLP from the inherently non-adiabatic measurements, where we identify data ranges that conform to (quasi)-adiabatic conditions. Each time interval of measurement that met a predetermined criterion was used to generate a value of SLP, and the mean from all estimates was selected as the estimated SLP. Despite the application of rigorous selection criteria, measured temperature data displayed variability at specific heating loads resulting in larger variance of calculated mean SLP values. Overall, the results show a linear dependence of the SLP with AMF frequency, as anticipated by current models. Conversely, measured amplitude-dependent SLP profiles of all studied constructs conform to no predictions of current models.

## Introduction

Magnetic nanoparticles (MNPs) have demonstrated utility in biomedicine due to their responsiveness to magnetic fields and generally favorable biocompatibility^1–5^. Magnetic nanoparticles have also demonstrated utility as hyperthermia agents to treat prostate cancer^6^ and recurrent glioblastoma multiforme, achieving regulatory approval in 2010 for the latter indication^1, 2, 7^. Heat is a potent anti-cancer therapeutic agent, particularly when it is combined with ionizing radiation^8^. When exposed to alternating magnetic fields (AMFs), magnetic nanoparticles can generate heat that is deposited locally^9^ to effect treatment; therefore, accurate methods to measure the heating are required.

The heating efficiency can be defined as the thermal power per unit mass dissipated by the magnetic material, or specific loss power (SLP)^10–16^. The term specific absorption rate (SAR) is often used interchangeably with SLP; however, its use may be confusing partly because regulatory agencies have adopted the terminology “Specific Absorption Rate” or “SAR” to define the rate at which power is absorbed by a volume of dielectric material, such as biological tissue, exposed to radio frequency electromagnetic radiation (or other forms of energy such as ultrasound)^17^.

Despite continuing debate, calorimetry continues to be the most common method to estimate the SLP^11, 18, 19^. The method is deceptively straightforward: heat generated by a magnetic nanoparticle suspension when exposed to an AMF is related to loss power through measured temperature change. In practice, however calorimetry experiments are considerably more demanding of both equipment and experimenter to obtain accurate measurements of transferred energy^12, 20–22^. Furthermore, analyses that ignore the inherent experimental variance may lead to erroneous conclusions that observed changes of SLP result from complex nanoparticle physics such as aggregation or inter-particle magnetic dipole interactions, when in fact, the observed changes may be due to inherent variance in the measurement methods. It is an objective of the current work to evaluate the extent of variance or ‘error’ inherent in the measurement itself. It has become common practice to infer information of the underlying nanoparticle structure or physics from these calorimetry or SLP measurements alone without further measurements that simultaneously query other physical properties of the magnetic colloids while exposed to time-varying magnetic fields, at the same conditions (i.e. AMF amplitude and frequency) of the SLP measurements.

The validity of calorimetry rests upon two fundamental assumptions, based upon the conservation of energy, which hold for AMF-driven magnetic nanoparticle heating: 1) The system is closed (no energy or mass exchange with external environment); and, 2) The work done by the system is solely of magnetic nature^23, 24^. If the process is adiabatic and no physical or chemical changes occur in the samples, except heating, then the SLP can be explicitly related to the measured heating by:

1 $$SLP=\frac{C}{m}\frac{{\rm{\Delta }}T}{{\rm{\Delta }}t}$$

where *T* is measured temperature, *t* is time elapsed, *C* is the heat capacity of sample (i.e. nanoparticles and suspending medium), and *m* is mass of nanoparticle sample, or magnetic material^12, 25^.It is common practice to use Eq. 1 for SLP measurements; however, this relation is explicitly correct *only if* the underlying assumptions are valid in the experiment.

The criterion of adiabatic conditions is fundamental in the thermodynamic sense, but it is experimentally unachievable because some temperature-dependent energy (heat) transfer always occurs across the sample-environment boundary. ‘Effectively’ adiabatic systems are attainable, but difficult to construct^14^ and they require elaborate thermal insulation (vacuum environment) and radiation shielding, with advanced materials to minimize heating from induced (electromagnetic) eddy currents, or ‘active’ heating of the shield to match internal sample temperatures to minimize heat transfer with external environment^11, 25^. Such systems are technologically complex and expensive and they inevitably limit the accessible range of magnetic field and frequency combinations because system component heating increases substantially with applied power^25^.

Consequently, most studies are performed using simple non-adiabatic devices^12, 14^, and there is currently no consensus on ‘best practice’ to measure temperature and calculate SLP. The underlying thermodynamic principle of adiabatic conditions defines the validity of Equation 1, yet it is the individual solutions developed to overcome technical and experimental challenges of ‘AMF-calorimetry’ that leads to varied experimental configurations and practices, some of which violate the underlying assumption(s)^11, 12, 14, 20, 21, 26^.

It is believed that errors can be limited by limiting the range of temperatures for the SLP calculations, which typically assume constant *C*. It is thus common practice to estimate the temperature increase per time step $\frac{{\rm{\Delta }}T}{{\rm{\Delta }}t}\,\,$from the initial slope of the temperature-versus-time curve^11, 12, 14, 20, 21, 27^. The rationale employed assumes that at the onset of heating, a) adiabatic conditions prevail making thermal losses to the environment negligible, b) temperature variations within the sample are also negligible; and, c) constant temperature approximations of heat transport properties produce negligible errors^12, 25, 27^. These assumptions are typically not tested or evaluated for each data set within a series of measurements^12, 16, 21, 26, 28, 29^. Bordelon *et al*. demonstrated that non-linear temperature rises can occur during the initial heating interval^12^, invalidating those initial data points for SLP calculation. Similarly, Wang, *et al*., note fluctuations due to thermal mixing and other sources occur at the onset of heating^21^.

A variety of calculation methods have been developed to estimate SLP from heating data^11, 14, 21^. Examples include linear fitting of the first 10 to 100 s, polynomial fitting and determination of the maximum slope, and numerical derivatives of the temperature-versus-time curve to calculate the temperature increase per time step^11, 12, 14, 26^. Recently, other non-adiabatic calculation methods have emerged based on the phenomenological Box-Lucas equation^30^, the assumption of linear (heat) loss^14^, or by means of a thermodynamic model that takes the heat exchange between the sample and the surroundings into account^31^.

Equipment and experimental conditions also contribute to variance. Volume and shape of the sample, material construction of container(s) placed within the inductor, and temperature measuring device(s) all contribute as potential sources of error. Temperature distribution within the sample is typically non-uniform due to convection, heat losses, and AMF field non-uniformities^20^. These conditions violate the fundamental assumptions of calorimetric methodology, making it necessary to perform a detailed analysis of the sources of error or variance^12, 20, 21^, and to perform calorimeter calibration^15^.

Differences in measurement methodology arising from the varied approaches to solve technical challenges associated with calorimetry in an induction coil further compound the difficulties to harmonize experimental results with theoretical predictions. Specifically, the magnetic nanoparticle suspension (sample) is placed into a container or vessel, which itself is placed into an insulated chamber that is surrounded by an induction coil. The induction coil geometry is typically a simple solenoid constructed from copper tubing through which coolant flows to compensate for coil overheating resulting from the electric current load and by EM self-induction. The field produced by simple solenoids is inhomogeneous, and the magnetic flux density can vary depending upon the magnetic properties of the sample creating a complex coupled magnetic hysteresis response. Furthermore, variations or fluctuations of electric current flowing through the coil can induce additional variations of heating from nanoparticles. Electronic instabilities in the power supply, capacitance network, and coil can produce variations in the sinusoidal current leading to generation of harmonics that often go unnoticed because they do not compromise performance of the device, yet they may contribute to generation of variance of heating response in the sample. Temperature measurements are typically performed with a single-point temperature probe that is most commonly constructed from optical fiber. Less common are bimetallic thermocouples because the inherent electrical interferences and potential for heating by induction, and infrared thermometry because of issues with emissivity and thermal profile at depth of sample^22^.

Performing SLP estimations in a large range of magnetic field amplitude and frequency variations is therefore exceedingly challenging, but such measurements can yield valuable information about the nanoparticle samples^12, 13, 32^. Varied heating behavior among magnetic nanoparticles often manifests only when measured through a broad range of fields^12^, rendering the many heating experiments performed at a single or a narrow range of field amplitudes incomplete^32^.

In this work, we present results of specific loss power measurements using four magnetic nanoparticle constructs that have been extensively characterized in previous studies^28, 29, 32^. SLP was estimated from data obtained in a range of frequencies (150–375 kHz) and magnetic fields (4–44 kA/m). We developed an estimation method that extends the approach presented by Bordelon *et al*.^12^. Central to this method is the identification of all time ranges in a single heating experiment that exhibit (quasi)-adiabatic heating conditions, generating an SLP distribution for each experiment. This approach enables study of inherent variation of SLP for each sample, within the measurement itself. Furthermore, our results of frequency- and amplitude-dependence of the SLP are compared to current models and highlight the necessity of a more accurate theoretical framework to explain the heating mechanism of magnetic nanoparticles.

## Results

**Figure 1:**
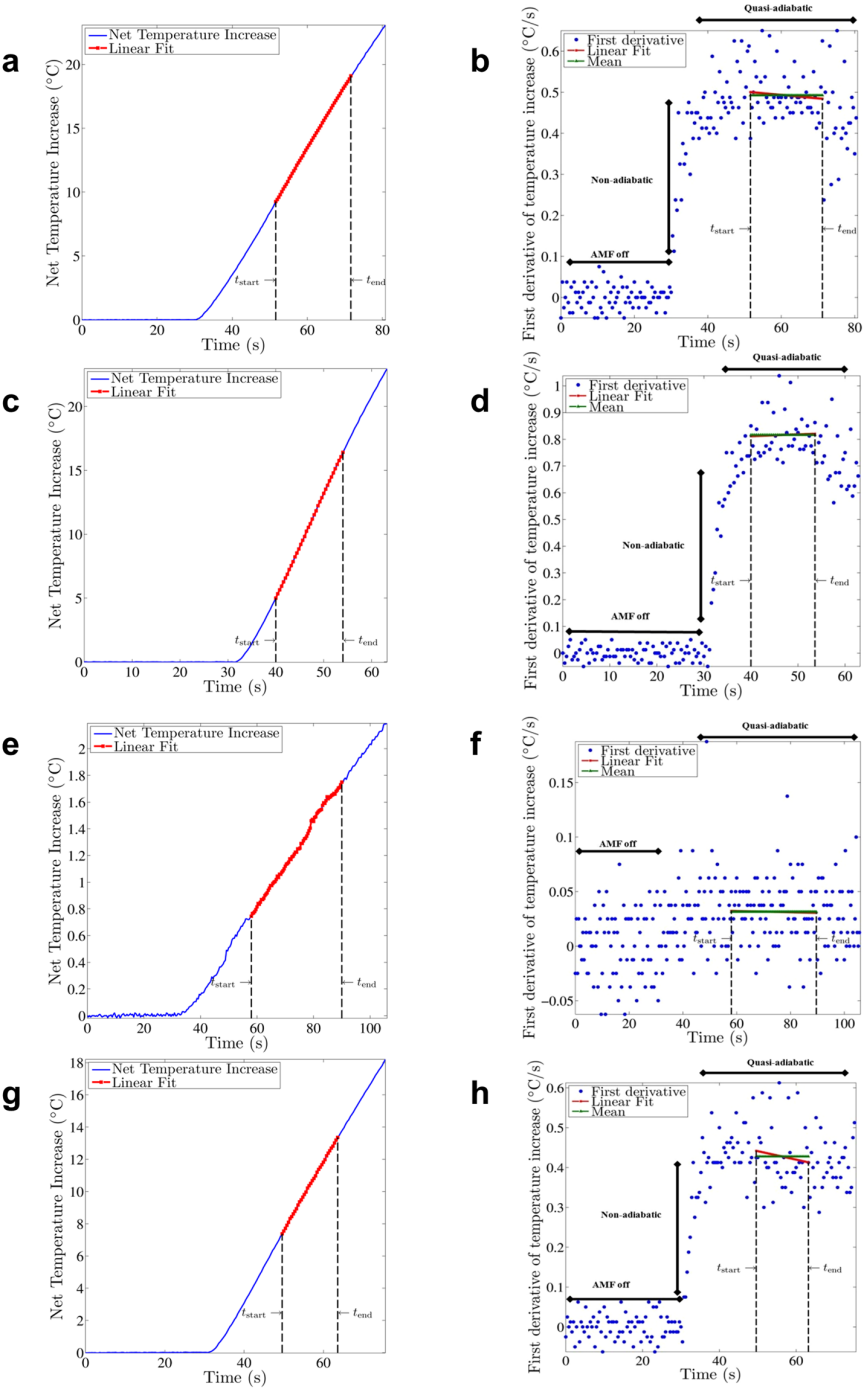
Sample of measured temperature rise data obtained from BNF-Dextran nanoparticles with applied AMF having amplitude 20 kA/m and at frequency 150 kHz. Estimation of SLP from heating rate data requires that (quasi-)adiabatic criteria are met, manifest by linear region of time-temperature curve. A least squares fit of the net temperature increase (sample temperature rise – water blank temperature rise) is performed in a chosen time range (t_start_, t_end_) (**a**). Simultaneously, the mean value and the y-intercept of a least square fit to the incremental net temperature change per time step are determined in the same time range (**b**). In case these values are within 5% of the net temperature rise slope, the heating in the chosen time range is considered to be (quasi)-adiabatic and this net temperature rise slope is used to calculate the SLP^12^. This procedure is repeated for every possible time range and every time range satisfying the criterion from which SLP values are calculated. In this study, the average of all obtained values that meet this criterion is reported as the measured SLP. Analogous figures are presented for JHU nanoparticles (**c**,**d**), nanomag^®^-D-spio nanoparticles (**e**,**f**), and MnFe_2_O_4_ nanoparticles (**g**,h).

Aqueous suspensions of BNF-Dextran, nanomag^®^-D-spio (both from micromod Partikeltechnologie GmbH, Rostock, Germany), and JHU (NanoMaterials Technology, Singapore) iron oxide magnetic nanoparticles (MNPs) were used. BNF and nanomag^®^-D-spio iron oxide nanoparticle constructs were coated with dextran; whereas JHU nanoparticles were citrate-stabilized. We also considered manganese-ferrite nanoparticles surface-coated with citrate^29, 33^. All magnetic nanoparticle constructs have been previously characterized^28, 29, 32, 33^. Their physical properties are summarized in Table 1. Heating data were obtained by methods described in Methods below. Representative corrected heating data, their first derivative (AMF amplitude 20 ± 1 kA/m (peak) and frequency 150 ± 10 kHz) are displayed in Fig. 1.

**Table 1:** Parameters of studied nanoparticles.

Name/Manufacturer	Description	Iron concentration (mg/ml)	Surface/Solvent
BNF-Dextran (84-00-102)/micromod^12, 28, 32, 53^	Multi-crystallite dense iron oxide core, polymer shell.	15 ± 2	Dextran/H_2_O
JHU/NanoMaterials Technology^32^	Multi-crystallite dense iron oxide core, surfactant shell.	90 ± 10	Citrate/H_2_O
nanomag^®^-D-spio (79-00-201)/micromod^32, 54^	Multi-crystallite diffuse iron oxide with polymer matrix	5.0 ± 0.8	Dextran/H_2_O
MnFe_2_O_4_/custom made^29^	Multi-crystallite dense iron-manganese oxid core, surfactant shell	11.3 ± 1.5	Citrate/H_2_O

Inspection of BNF nanoparticle heating data (Fig. 1a) reveals an apparent linear temperature rise at onset of AMF power, *t* = 30 s; however, the first derivative of heating curve (Fig. 1b) displays three distinguishable regions of rate of rise. In the first, occurring at time *t* = 0 to 30 s, the AMF power is off and *dT/dt* = 0 °C/s as expected. Variance is evident with values ranging between about −0.05 to about 0.07 °C/s. We can infer from this that a minimum heating rate of ~0.1 °C/s can be reliably measured. Lower than this, and we risk interpreting data that is indistinguishable from the ‘baseline equipment uncertainties’. At onset of heating, a sharp rise of heating rate, from 0 °C/s to ~0.5 °C/s, is observed that persists for several seconds (*t* = 30 s to about 40 s). There appears no plateau and the transition is abrupt at onset, and at its conclusion when heating rates enter the third region (*t* = ~40 s to 80 s). Defined (quasi)-adiabatic criteria (see Methods) for SLP estimation were applied to data in this latter region. Several possible combinations met the criteria, precluding an objective selection for a unique set. One possible choice of points, presenting a 20-s range (*t* = 52 s to 72 s) is shown. It is noteworthy, however that the observed variance ranges stochastically from >0.2 °C/s to >0.6 °C/s through the entire ~40 s to 80 s of heating. It was therefore necessary to conduct a comprehensive statistical analysis of the entire range of possible combinations of data meeting the defined (quasi)-adiabatic criterion. Heating data obtained from the other nanoparticle samples using the same magnetic field conditions demonstrates that these features appear generally for all nanoparticle constructs tested, but in some cases (e.g. nanomag^®^-D-spio) the steep ascent of rate of rise is much less prominent and of much shorter duration (see Fig. 1c–h).

**Figure 2:**
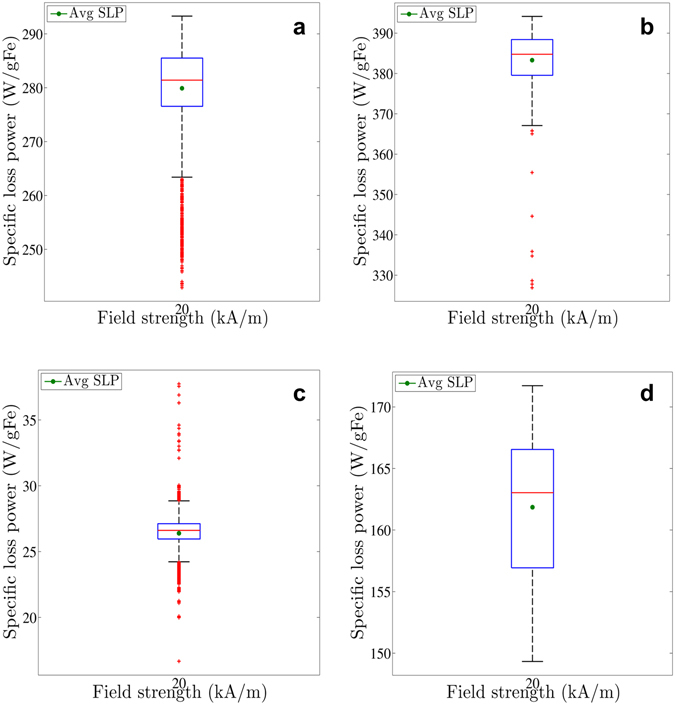
Box-and-whisker plot of all possible SLP values that meet the (quasi-)adiabatic criterion in the case of BNF-Dextran (**a**), JHU (**b**), nanomag^®^-D-spio (**c**) and MnFe_2_O_4_ (**d**) nanoparticles measured at 150 kHz and 20 kA/m. The (green) dot within the box always denotes the mean of all SLP values corresponding with (quasi)-adiabatic heating. The blue lower and upper box boundaries are always the first and third quartiles (SLP_25%_ & SLP_75%_), whereas the median (SLP_50%_) is depicted by the red horizontal line. The difference between the end of the whiskers (adjacent values) and the box boundaries can maximally be 1.5 times the interquartile distance, i.e. SLP_75%_ - SLP_25%_. The red crosses represent each calculated SLP value lying outside the range between the whisker ends, i.e. outliers.

We estimated SLP using methods described below (see Methods). Figure 2 depicts box-and-whisker plots of the calculated SLP corresponding to all possible combinations (time frames) meeting the (quasi)-adiabatic criterion. All measurements reported in Fig. 2, demonstrate representative results and were performed at 150 kHz and 20 kA/m. Full analysis was similarly conducted for measurements at all other field amplitude and frequency combinations (data not shown). Measured heating rates and mean SLP values obtained vary significantly among all tested nanoparticles, consistent with previous reports^12, 29, 32^. Despite the restriction limiting choices to combinations of data meeting the quasi-adiabatic condition, variance remained significant. Therefore, *both* the mean SLP (green dot) *and* the inherent variance contain information and must be considered in a complete analysis of SLP. In Table 2, we report additional measures of the variance.

**Table 2:** Summary of variation of SLP measurements at 150 kHz and 20 kA/m.

Nanoparticle solution	Average SLP (W/gFe)	Standard deviation (W/gFe)	Standard deviation/Average SLP (%)	IQR (W/gFe)	IQR/Average SLP (%)	Inter-adjacent distance (W/gFe)	Inter-adjacent distance/Average SLP (%)	Maximum-minimum (W/gFe)	(Maximum-minimum)/Average SLP (%)
BNF-Dextran	280	9	3	8.90	3.18	29.90	11	50.40	18
JHU	383	7	2	8.80	2.30	27.00	7	67.20	18
nanomag^®^-D-spio	26	1	5	1.16	4.40	4.63	18	21.06	80
MnFe_2_O_4_	108	4	3	6.44	5.94	14.99	14	14.99	14

Note: In addition to average SLP, additional measures of variation are provided - standard deviation, interquartile range (IQR), inter-adjacent distance, and the difference between the maximum and minimum calculated SLP values. The ratio of these different measures of variation and the average indicate a relative inherent variance to estimated SLP. SLP of BNF-Dextran, JHU, and nanomag^®^-D-spio nanoparticles is reported as W/(g Fe), whereas for the MnFe_2_O_4_ nanoparticles it is reported as W/(g magnetic material).

BNF-dextran, JHU, and MnFe_2_O_4_ (See Fig. 2a,b,d) exhibit a lower value of the standard deviation of their mean (2–3%) than nanomag^®^-D-spio (5%) (Fig. 2c). Comparison of SLP with the interquartile range (IQR) and the inter-adjacent distance (see Table 2) among the particles denotes a possible pattern that higher SLP values coincide with decreased variance. On the other hand, the lower heating-type nanoparticles nanomag^®^-D-spio, display a relatively large number of outliers above and below the whiskers increasing the uncertainty of the SLP estimations (see Fig. 2c).

**Figure 3:**
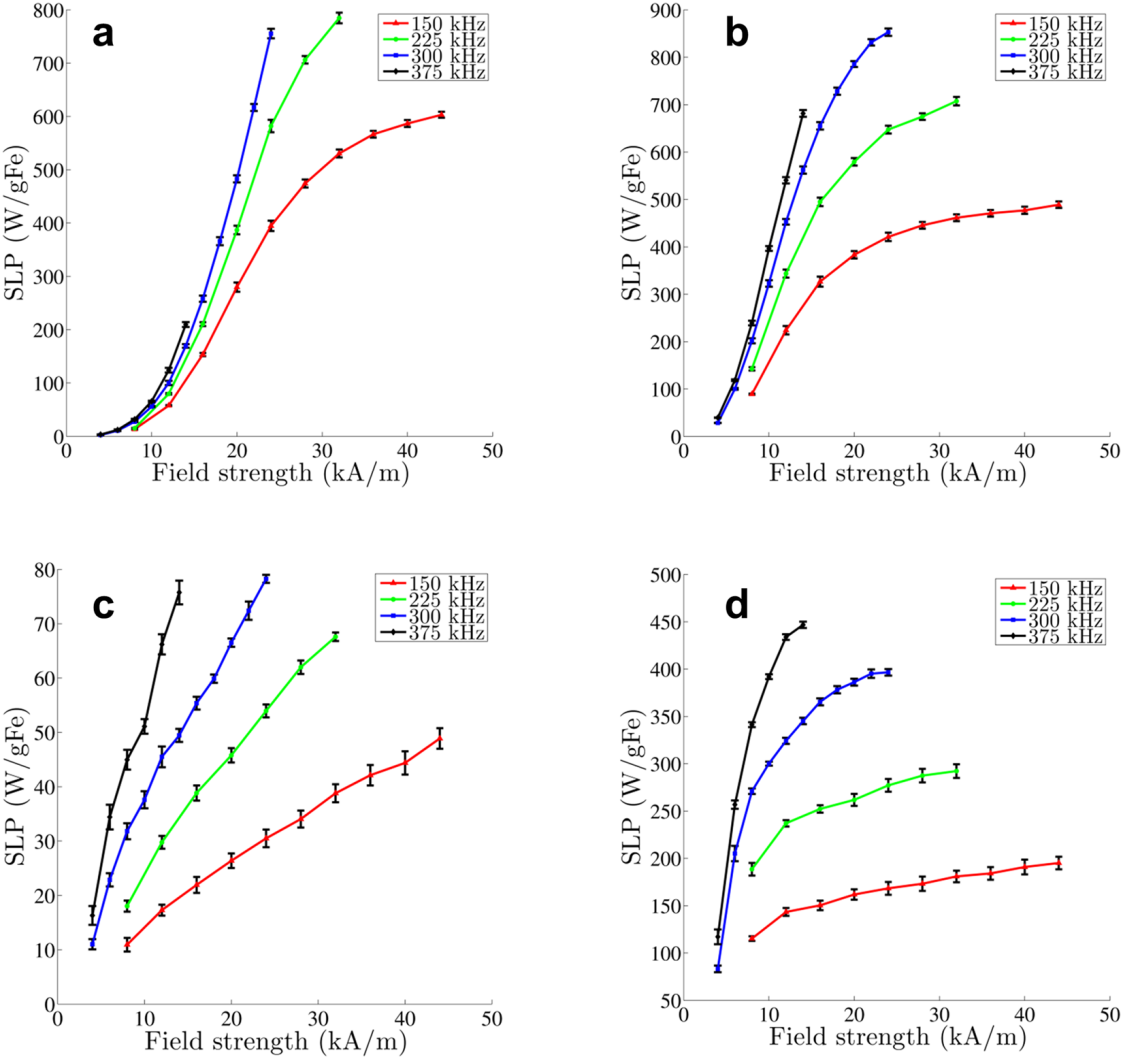
Specific loss power (SLP) measurements versus field strength of four different magnetic nanoparticle solutions (**a**: BNF-Dextran, **b**: JHU, **c**: nanomag^®^-D-spio, d: MnFe_2_O_4_) at four different frequencies ranging from 150 kHz to 375 kHz. Only the mean SLP value is reported, and the error bars are the standard deviation of all possible SLP values.

Amplitude-dependent SLP results are shown in Fig. 3. At 150 kHz, BNF-Dextran nanoparticles display low SLP at lower amplitudes, whereas the heating efficiency rises quickly at amplitudes between 15–20 kA/m (Fig. 3a). Saturation to 600 W/g Fe appears at ~40 kA/m. This ‘S’-shaped SLP curve is characteristic of the ‘hard ferrite-type’ materials^12, 29, 32^. This profile is also evident at 225 kHz, but at higher frequencies equipment limited achievable amplitudes.

JHU nanoparticles, on the other hand demonstrate higher heating efficiency at lower amplitudes than did the BNF nanoparticles (Fig. 3b). Considering the 150 kHz experiment, the SLP measured at 28 kA/m is within 10% of the maximum SLP measured at 44 kA/m. Apart from measurements at 375 kHz, where we observed no evidence of saturation, the same trend is present at all lower frequencies tested.

Nanomag^®^-D-spio nanoparticles displayed lower thermal dissipation than all other nanoparticles studied, with a maximum measured SLP value of 78 W/g Fe at 300 kHz and 24 kA/m. At 150 kHz, the field amplitude-dependent SLP appeared to be linear. At higher frequencies, an inflection point is hinted which suggests a deviation from the linear pattern. Saturation, evidenced by appearance of a constant SLP with amplitude, was not observed; although, measurements obtained from nanomag^®^-D-spio nanoparticles possessed large inherent variance precluding definitive conclusions (Fig. 3c).

MnFe_2_O_4_ nanoparticles displayed SLP(*H*) similar to JHU nanoparticles (Fig. 3d), exhibiting a rapid ascent of heating rate at low field amplitude, consistent with the ‘soft-ferrite’ type of particle^29^. At 375 kHz an inclination towards a plateauing of SLP with amplitude, indicating possible saturation, was observed for the MnFe_2_O_4_ nanoparticles, whereas the JHU and BNF-Dextran nanoparticles did not display similar behavior at this frequency. Thus we may infer that MnFe_2_O_4_ nanoparticles may realize saturation at lower field amplitudes than their JHU counterparts.

**Figure 4:**
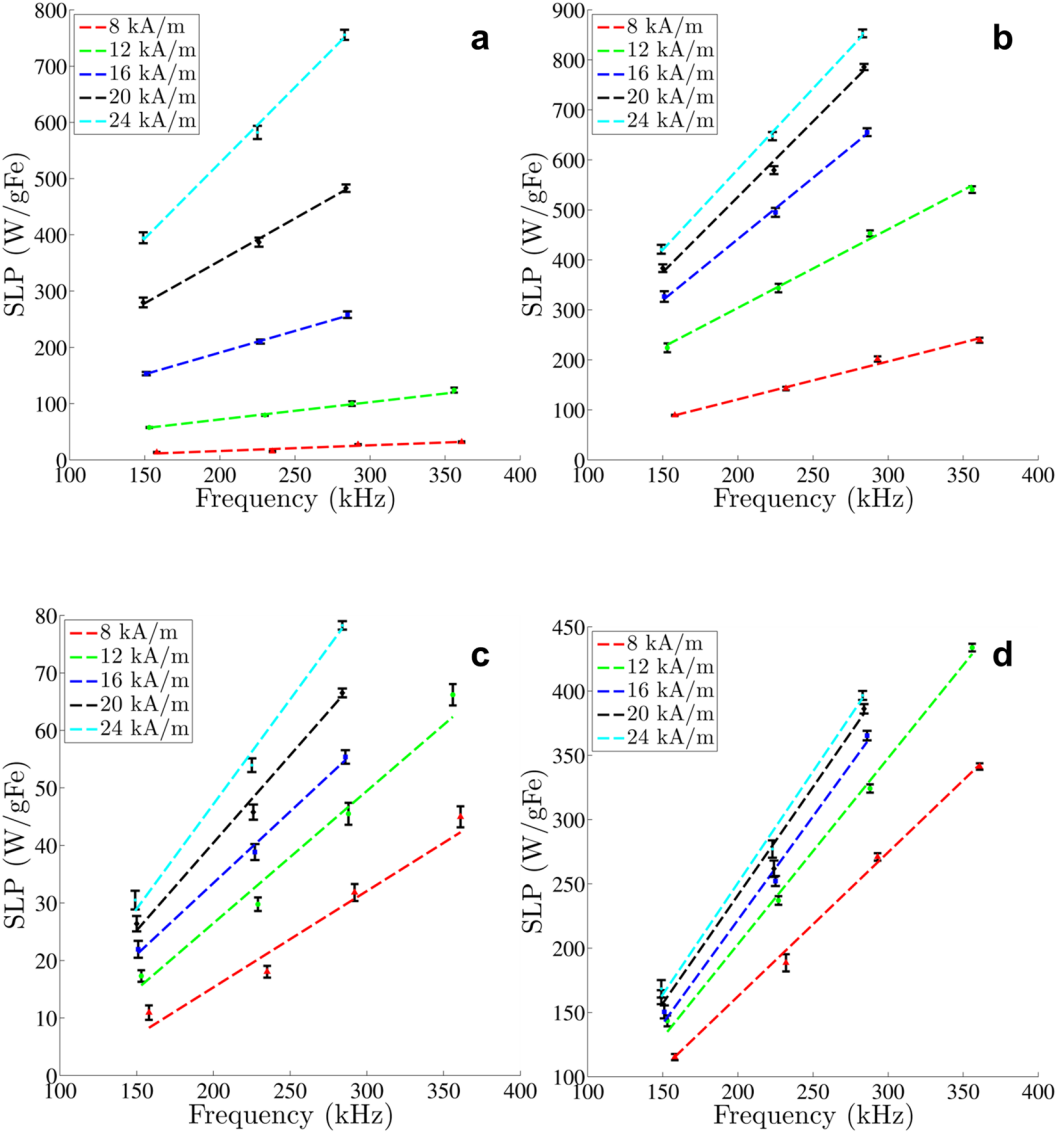
Specific loss power (SLP) measurements versus frequency of four different magnetic nanoparticle solutions (**a**: BNF Dextran, **b**: JHU, **c**: nanomag^®^-D-spio, **d**: MnFe_2_O_4_) at five different field strengths ranging from 8 kA/m to 24 kA/m, in increments of 4 kA/m. Data shown represent the average SLP value with the standard deviation as error bars.

The frequency-dependence of SLP values obtained from all nanoparticles for frequencies 150 to 375 kHz at field amplitudes ranging from 8 to 24 kA/m are displayed in Fig. 4. For all amplitudes tested we performed a weighted-linear-least-squares fitting of the SLP vs frequency. BNF-Dextran and JHU nanoparticles demonstrated a linear frequency-dependence of their SLP in the studied range. Estimates of SLP from nanomag^®^-D-spio and MnFe_2_O_4_ nanoparticles also display the linear trend, although the measured variance was greater (Fig. 2c and d and Table 2).

## Discussion

Magnetic iron oxide nanoparticles have garnered significant interest in recent decades for their applications in medicine. In magnetic hyperthermia, magnetic iron oxide nanoparticles generate heat when they are exposed to an alternating magnetic field, providing utility for cancer therapy. Accurate measurement of the heating potential, or SLP, of the particles is critical as the heat generated is the therapeutic agent. Estimation of the SLP from a specific magnetic iron oxide nanoparticle sample is conceptually straightforward, because the SLP can be calculated from direct calorimetric measurements of heating using specific conditions. In practice, however SLP determination is considerably more complex because the inherent thermodynamic assumptions upon which these measurements are founded present significant technical and technological challenges. The transformation of measured heating rate data into a measure of energy produced requires strict adherence to adiabatic criteria, which is typically not satisfied in experimental situations.

Thus it is the case that in practice, measurement of magnetic nanoparticle heating is complex because both electromagnetic and thermal processes contribute to heating, measured temperature, and the associated measurement errors. Furthermore, suspensions of magnetic colloids represent an ensemble collection of particles having a distribution of magnetic and structural properties; and, they exhibit complex magnetically-driven responses when subjected to forced hysteresis by an alternating magnetic field, particularly when field amplitude and frequency are varied. Significant effort has been devoted to development of physical models that describe the time-dependent relaxation properties of fine-particle magnetic systems, yet the complexity of these systems continues to elude unifying descriptions^16, 29, 32, 34^.

Despite numerous theoretical attempts^16, 30, 34–37^, our understanding of the heating mechanism(s) resulting from the time-dependent relaxation of magnetic nanoparticles in magnetically-driven fields is limited. It is widely reported both from a theoretical and experimental point of view that interparticle magnetic dipole-dipole interactions impose a significant influence on the magnetic heating efficiency, yet comparisons of these predictions with experimental data continue to generate debate^29, 38–41^. The long-range nature of magnetic (and electrostatic) interactions can cause the formation of more complex structures, such as chains, rings or clusters, changing the overall specific loss power^28, 29, 41–46^. Structural measurements of magnetic fine particle colloids using *identical* experimental conditions as SLP measurements (i.e. AMF amplitude and frequency) have yet to be performed leaving a significant gap in experimental measurements. Furthermore, viscous losses^47, 48^ and the surface to core anisotropy ratio^34^ have a large influence on the heating efficiency. Attempts to measure SLP variation with nanoparticle aggregation or ‘fixation’ in high viscosity media typically neglect to account for changing thermal transport properties of the suspending medium ^20, 21, 24, 25, 27, 29, 31, 40, 45–48^. Finally, many magnetic iron oxide nanoparticles that have demonstrated utility for biomedical applications comprise a complex internal structure, often with ‘multi-crystallite’ or ‘multi-domain’ magnetic cores^16, 32^. The effective magnetic moment of these multi-crystallite core magnetic nanoparticles is affected by the intra-core inter-crystallite interactions^32, 49, 50^. In short, significant gaps in our theoretical and experimental capabilities limit our understanding and our ability to model the complexities of AMF-driven hysteresis heating displayed by colloidal suspensions comprising ensembles of magnetic fine particles^16^.

Through a varied range of the measurement conditions and estimation of the resulting SLP values, we note in the presented results that significant and variable experimental variance persists, potentially leading to erroneous conclusions if they are ignored. It is particularly noteworthy that the pattern of the variance seemingly coincides with the range of heating rates measured or estimated SLP values (Figs 1, 2 and S1). In other words, the heating rate of the particles under identical magnetic field conditions, apparently contributes to the extent and direction of the variance or error, relative to the mean. This variance, interestingly is neither consistent for a given nanoparticle construct measured at different conditions, nor is it consistent for different nanoparticles measured at the same experimental conditions. This implies a nanoparticle-specific heating contribution to the observed variance, i.e. structural or magnetic, but a conclusion cannot be determined on the basis of SLP measurements alone. Further structural characterization using the same conditions as for SLP measurements is warranted.

On the other hand, we might consider that the measured variances (including the frequency and value of outliers) among the multiple constructs indicate that the physical and geometric properties of the calorimeter and sample contribute significantly, perhaps dominate, the observed measurement errors^21^. If true, this suggests that compensating for the non-adiabatic conditions, while necessary, is insufficient to wholly account for variability and reduce uncertainties of the measured values. Furthermore, inspection of the variance with a focus on range of heating rate (see Figures S1a–d) reveals no consistent pattern of variance depending upon heating rates. Thus, even within a set of measurements performed consistently with the same equipment, the inherent uncertainty can be unacceptably high (>10%, Figs 2 and S1), making it challenging to conclude that changes of SLP are strictly due to changing structural or magnetic features of the magnetic colloid, or manifestations of properties of the calorimeter^12, 29, 32^. Our analysis of the data suggests that comparisons utilizing SLP measurements to infer physical or magnetic properties of the nanoparticles must be accompanied by a detailed analysis of the inherent variances in the measurements to place limits on the reliability of the observed trends, *and* additional comprehensive measurements using multiple orthogonal techniques must be employed^32^.

A widespread and underestimated source of error is the inhomogeneous magnetic field produced by simple solenoids–a most common apparatus^20^. It is possible to modify a solenoid to obtain reasonably homogeneous flux densities within a defined volume suitable for SLP measurements^51^. Even with use of an induction coil producing a homogeneous field within the sample volume, as in the present work, an inhomogeneous temperature distribution within the sample is expected due to inherent distribution of nanoparticle magnetic and structural properties^20, 21^. Inhomogeneity of temperature within the sample can also be attributed to the sample geometry and volume^21^. Sample volume and geometry used in the current study were kept constant, with measured samples of 1 g, and thus the height of the magnetic fluid in the tube was constant for all samples. Equilibration of the sample with the environment before the heating experiment is crucial to avoid slow cooling or heating due to the environment, and correcting for solvent (water) blank heating is necessary, and a potential source of variance^12^.

Solvent heating can potentially arise from eddy currents induced in conductive media when alternating magnetic fields are applied, and from coil heating or calorimeter effects. Subtracting solvent blank temperatures at each set of experimental conditions from the nanoparticle temperature measurements effectively corrects for calorimeter heat capacity and effects of power absorption on calorimeter and media, yet it presents an additional source of variability^12^. Finally, limiting the duration of the SLP measurement (<100 s in our experiments) minimizes measurement uncertainty^20, 21^ and potential degradation of the nanoparticles due to a long exposure to magnetic fields, especially when studying a wide range of fields and frequencies.

The analysis method itself can also influence estimated SLP. With SLP estimation from the initial-slope method, it is assumed that the sample temperature is homogeneous and that heat losses are negligible when hysteresis heating commences^11, 12, 14^. It is also assumed (though rarely measured) that these conditions hold for some chosen duration during the measurement^14^. Wang *et al*.^21^ have identified increased uncertainty in SLP estimation accompanies long-duration heating, particularly at high amplitudes and heating rates resulting from increased rate of heat loss. Instead of assuming that heat losses are only negligible in the beginning, we searched the entire duration of the heating experiment for regions demonstrating negligible heat loss. Those quasi-adiabatic conditions produce a linear rate-of-rise and associated constant (non-zero) first derivative of the temperature increase, as illustrated in Fig. 1a–h. While a deviation from the linear pattern is not readily apparent in the temperature-time heating data (Fig. 1a,c,e and g), a transformation to the first derivative (following methods described by Bordelon *et al*.^12^) readily displays both time-dependent trends and inherent variances (Fig. 1b,d,f and h).

It is worth noting, at this point, that other methods, such as ‘symmetric difference’ (e.g. $[{\rm{\Delta }}{T}_{net}({t}_{n}+1)-{\rm{\Delta }}{T}_{net}({t}_{n})]/({t}_{n}+1-{t}_{n})$), may be used to generate the derivative with greater efficiency or convenience. Regardless of the method chosen, the point remains that the derivative of temperature-time curve offers reliable and precise mathematical analysis of the adherence to adiabatic criteria.

Before the onset of heating with the magnetic field, the nanoparticle solution is in thermal equilibrium (within the stochastic variance) with its surroundings, as evidenced by the first derivative that is zero. At the onset of heating, the first derivative steeply rises, indicating rapidly changing conditions that may arise from thermal mixing^12^. On the other hand, the steeply rising first derivative of the heating curve may indicate a rapid transition of heating rate that results from profound spatial rearrangements of nanoparticles in the solution in response to the driving external field. In other words, the nanoparticles may be forming structures in response to first application of the magnetic field that affect the heating. While analysis of the heating rate data alone cannot distinguish between thermal and magnetothermal effects, it has been demonstrated that many magnetic nanoparticles in suspension can exhibit complex colloid structures arising from inter-particle dipole-dipole interactions^28^, even at zero field (i.e. *H* = 0). When exposed to a field, i.e. at the instant the alternating field is applied to generate field-driven hysteresis heating, the spatial arrangement of the magnetic nanoparticles in the colloid changes in response to the field thus altering interparticle dipole-dipole interactions, leading to rapidly changing heating rates^29^. This possibility presents an interesting physical phenomenon worthy of further investigation, but one that would require considerable structural characterization at AMF conditions applied for SLP measurements. Such measurement capabilities were unavailable for the present work. Note also, that the degree to which the rapid rise of temperature manifests depends upon both nanoparticle type and concentration (Fig. 1)^12, 29^, suggesting possible physical-structural causes. Additional time-dependent magnetic and structural measurements are needed to ascertain the origin of this behavior.

**Figure 5:**
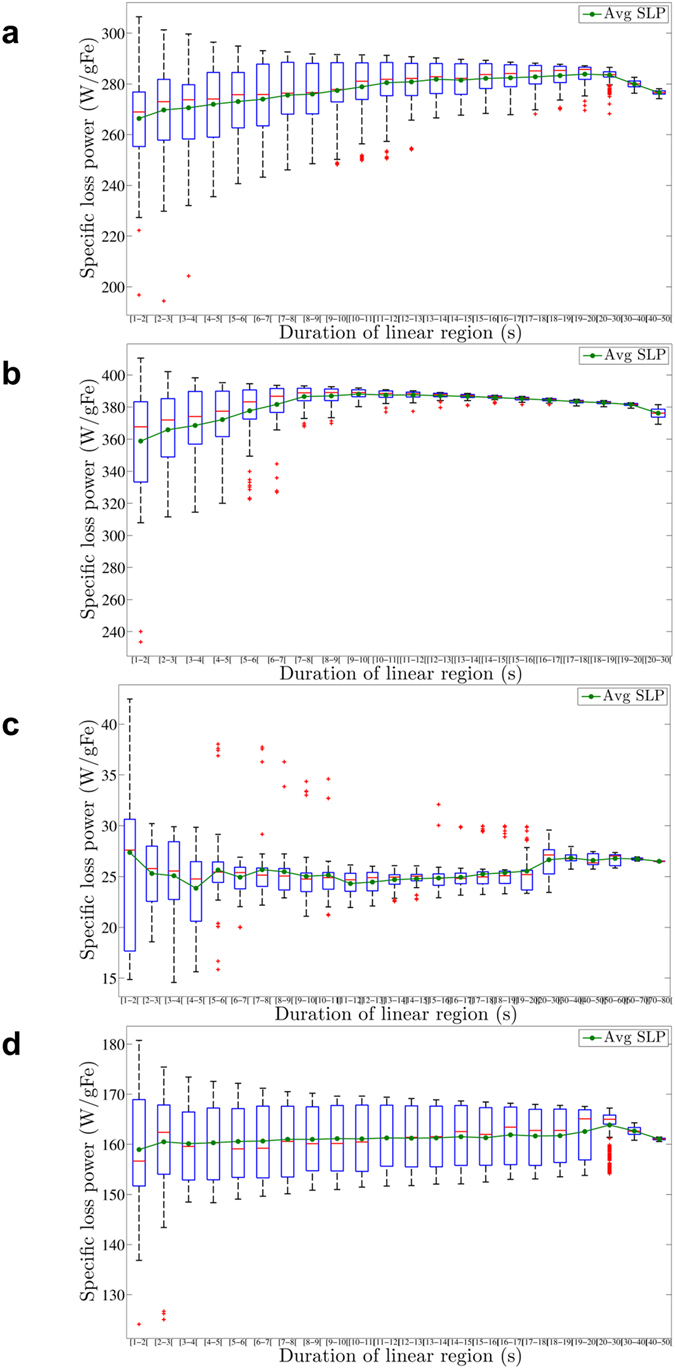
Possible SLP values, satisfying the (quasi)-adiabatic criterion, as a function of the time duration of the linear region (BNF-Dextran – 150 kHz and 20 kA/m (**a**)). The smaller the chosen duration of the linear region, the larger the variation in SLP values. To reduce this variation, only durations longer than 6 seconds are included in the calculation. The average SLP value (green line) and variation for each time duration larger than 6 seconds are similar (maximum 5% difference from [6s–7s] to [40s–50s]). Analogous figures are depicted for JHU (**b**), nanomag^®^-D-spio (**c**), and MnFe_2_O_4_ nanoparticles (**d**).

Regardless, it is clear that the first seconds of a heating experiment often fail to satisfy (quasi)-adiabatic criteria, and thus should not be used for SLP calculations. Afterwards, the first derivative plateaus to a constant (non-zero) value; however, this is accompanied by stochastic fluctuations arising from many potential sources. Therefore, many time frames within the plateau region demonstrate insufficient (quasi)-adiabatic behavior. When (quasi)-adiabatic conditions are met, however the slope of the temperature increase is expected to be equal to its first derivative. The slope of the temperature increase was determined by linear-least-squares fitting to the data. On the other hand, the mean value in a time regime, i.e. t_start_ to t_end_, provides an alternative and independent means to calculate the first derivative within specific time frame from t_start_ to t_end_. Mathematically, the *y*-intercept of a linear fit of the constant region of the first derivative of the heating curve is equal to the mean value of the points in that region, whereas the slope of the linear fit is precisely zero. Thus, when analyzing experimental data, we have the opportunity to apply multiple rigorous mathematical criteria as controls to ascertain consistency and relative error. We have applied the criterion proposed by Bordelon *et al*.^12^ that the three independent values obtained from analysis of the heating curves must be within 5% of each other within a certain time frame, to consider the time frame to be valid representation of (quasi)-adiabatic conditions. Roughly, this criterion corresponds to a 95% precision, as it requires a 5% consistency among the calculated values. Note that it is impossible to estimate accuracy because no reference standards are currently available for such measurements. By repeating this process for every possible time frame, we identified each (quasi)-adiabatic zone. The average of all possible SLP values, each corresponding to a different (quasi)-adiabatic zone, is considered to be the measured SLP value for that experiment. We analyzed each possible (quasi)-adiabatic time frame, albeit with a minimum duration of 6 s. In Fig. 5, we provide a representative set of analyzed data for all possible SLP estimates, meeting the set (quasi)-adiabatic criterion, as a function of the duration of the linear region for the nanoparticles tested with AMF at 150 kHz and 20 kA/m.

**Figure 6:**
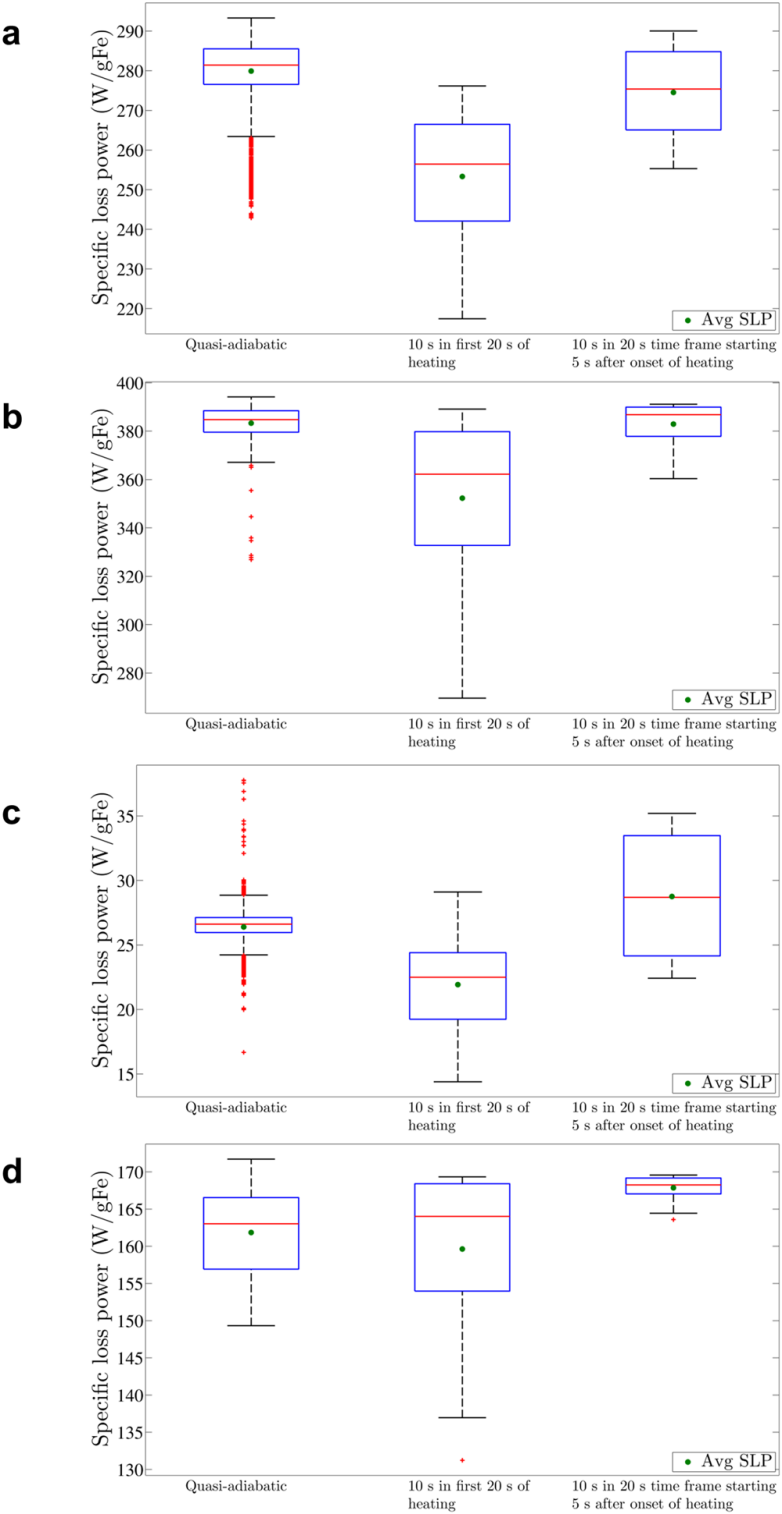
Box-and-whisker plot of SLP values obtained when applying different criteria to the same measurement performed at 150 kHz and 20 kA/m for BNF-Dextran nanoparticles (**a**), JHU nanoparticles (**b**), nanomag^®^-D-spio (**c**), and MnFe_2_O_4_ nanoparticles (**d**). The first criterion is our proposed (quasi)-adiabatic criterion, whereas the second represents all 10 s periods in the first 20 s of heating. The third criterion also includes every 10 s period, but in a 20 s timeframe starting 5 s after onset of heating.

The choice of criterion used to estimate the SLP significantly influences the resulting SLP estimation and more significantly affects the inherent variance. In Fig. 6, we compare the SLP estimation of the nanoparticle heating experiments at a representative field condition of 150 kHz and 20 kA/m by selected criteria. If one decides to use the initial-slope method and use a 10 s period in the first 20-s of heating, values between 220 and 275 W/g Fe can be obtained for BNF nanoparticles (Fig. 6a). When compared to our quasi-adiabatic criterion, the SLP values obtained using the initial-slope method are significantly lower. The trend of significantly lower SLP values persists for the other nanoparticle constructs as well (Fig. 6b–d). This suggests a highly inaccurate estimation arising from non-negligible initial heating fluctuations, suggesting that results obtained using similar criteria be re-examined. Additional comparisons with other criteria are provided in Supplementary Materials, Figures S2a–d. Avoiding the first five seconds of heating (non-adiabatic, and where potential thermal mixing is present, as visualized in Fig. 1b,d,f and h), helps to generate SLP values closer to the quasi-adiabatic criterion. Using this criterion, the average SLP for BNF-Dextran and JHU nanoparticles is comparable to the quasi-adiabatic result (<2% difference). Average SLP values for MnFe_2_O_4_ and nanomag^®^-D-spio nanoparticles are respectively 3.7% and 9% higher than their quasi-adiabatic counterparts. Interestingly, this criterion has associated an interquartile range greater than in the quasi-adiabatic case for all particles studied, except for MnFe_2_O_4_ nanoparticles (Fig. 6b,c).

Arbitrary choice of a single time interval to estimate the SLP thus introduces a high likelihood to generate inaccurate and unreliable results. Multiple different SLP values can be obtained, when using such single-modality fixed criteria due to the inherent variance, neglect of heat generating variations in the sample, and heat transfer properties in the apparatus. This offers an explanation for the significant variability of estimations of the SLP observed throughout the literature^11, 14^. To harmonize SLP estimation, we considered every possible time frame and calculated the average value among several possible outcomes for each region that satisfied the quasi-adiabatic criterion. Thus we achieved a higher precision, and we also gained information about the inherent variance. Note this variance represents the inherent variance measured within a single (each) measurement for the specific measurement conditions employed (i.e. sample, calorimeter, field-frequency combination, heating rate, etc.). Replicate, i.e. three or more independent repeat measurements using a given sample at same conditions were also performed. Also performed were ‘comparative repeat’ measurements using separate (virgin) samples prepared from the same parent stock of nanoparticle suspension. These variations of replicate and comparative repeat measurements were performed for each of the four nanoparticle materials studied. Given that each heating rate measurement produced multiple data points, each corresponding to multiple analyzed time regions, a rigorous statistical analysis of data obtained from each measurement session provides a useful and robust assessment of inherent measurement variance for the specific experimental conditions which reasonably estimates the repeatability or the precision of the measurement at those conditions. By contrast, replicate measurements provide information that can yield insights into reproducibility of methods and of sample or equipment stability, particularly upon cycling through AMF field exposure. Inconsistent experimental methodology or changing nanoparticle properties, e.g. nanoparticle aggregation, is expected to produce different heating behavior with successive measurements that would manifest as significant deviation (i.e., above the individual measurement variance described above). Such deviations were not observed, which along with no observed precipitates forming in the sample containers, rules out the possibility that agglomeration or physical aggregation was the source of the observed variances. The formation of transient and dynamic magnetically-driven higher-order structures however cannot be ruled out from the SLP data as previously discussed. It is noteworthy that replicate (or repeat) measurements numbering fewer than ~10 are typically considered insufficient for analysis with descriptive statistical methods^52^. In all cases for which replicate and comparative repeats were measured, average SLP values were obtained with a deviation of 2% and with overlapping distributions, suggesting that both methodology and sample properties were consistent. Nonetheless, these repeats do not give the same information about the accuracy of a single measurement, i.e. the inherent variance of a single measurement which may indicate subtle (reversible or dynamic) changes within particles or equipment during measurements.

In conclusion, we report results obtained from amplitude- and frequency-dependent SLP measurements of four magnetic nanoparticle constructs. The developed estimation method identifies all time ranges of the heating experiment that satisfy quasi-adiabatic conditions. The quasi-adiabatic criterion is a useful tool to study the SLP in a wide range of frequencies and fields, because it is mathematically consistent with the underlying thermodynamic assumptions of calorimetric methods used to transform time-temperature heating data into energy absorbed by the suspending medium. In our analysis, we explored the contributions of individual quasi-adiabatic time ranges that correspond to a specific SLP value. Consequently, we obtained a distribution of possible values for each heating experiment, providing valuable information about the non-negligible inherent variance of SLP measurements. We propose these (or similar) methods be applied to measured data obtained from nanoparticle heating experiments, regardless of equipment configuration. Ascertaining the validity of measured trends of SLP values with varied experimental conditions, e.g. magnetic field, concentration, particle aggregation, therefore requires a rigorous analysis of the inherent variance to determine the limitations of reliability of each data point taken within the measurements. With rigorous application of these criteria and developed methods of analysis, we demonstrate frequency and amplitude dependence of the four nanoparticle constructs to be qualitatively different, providing interesting examples for further study.

## Methods

### Magnetic nanoparticle solutions

In this study, we used four different aqueous nanoparticle suspensions: BNF-Dextran, JHU, nanomag^®^-D-spio and MnFe_2_O_4_ nanoparticles. BNF-Dextran and nanomag^®^-D-spio’s were obtained from micromod Partikeltechnologie GmbH (Rostock, Germany), while JHU nanoparticles were provided by NanoMaterials Technology (Singapore). The MnFe_2_O_4_ nanoparticles on the other hand were custom-made^29^.

BNF-Dextran nanoparticles consist of a dense core of multiple parallelepiped iron oxide crystallites, formed by a high temperature and pressure homogenization process, coated by dextran^32, 53^. JHU nanoparticles on the other hand are synthesized by a high gravity controlled precipitation, again forming a dense core of multiple spherical iron oxide crystallites, after which they are coated with citric acid^32^. Nanomag^®^-D-spio’s are formed by coprecipitation of iron salts in the presence of dextran, resulting in a diffuse core with spherical crystallites dispersed in a dextran matrix^32, 54^. Finally, MnFe_2_O_4_ nanoparticles are synthesized by the coprecipitation method and surface-coated with citric acid^29^.

### Alternating magnetic field (AMF) equipment

The alternating magnetic field equipment, as previously described in^51^, comprises three main components: the power supply, an external capacitive network and a vertical solenoid induction coil. The ensemble creates a resonant circuit. During each experiment, the coil is cooled using a closed-loop circulating water system. The four-turn copper coil inductor (length: 102 mm, inner diameter: 52 mm) is specifically designed to generate and sustain a homogeneous magnetic field (<±10% peak amplitude) having a cylindrical geometry within the inner volume of the coil (~125 cm^3^). Other design elements further enhance performance over standard solenoid coils^51^. Before every experiment, the power supply voltage was mapped to a measured magnetic field amplitude by means of AC field probes (AMF Life Systems, Inc., Auburn Hills, MI)^51^. The solenoid coil itself has been calibrated with copper wire^15^. The magnetic field amplitude at fixed frequency can easily be altered by modifying the power supply voltage. The frequency was adjusted by changing the capacitance of the external capacitive network. The range of attainable frequencies was 150–375 kHz. The range of accessible magnetic field amplitudes of the equipment depends upon the frequency. At 150 kHz, we performed heating experiments at 4 kA/m intervals between 8 and 44 kA/m. Amplitudes from 8 to 32 kA/m with a 4 kA/m interval were achieved at 225 kHz. At 300 kHz, the magnetic field strength was varied with steps of 2 kA/m between 4 and 24 kA/m. Finally, we were able to perform measurements every 2 kA/m between 4 and 14 kA/m at 375 kHz.

### Experimental setup for specific loss power (SLP) measurements

The specific loss power of nanoparticle sample is defined as the measured thermal loss power, normalized by mass of magnetic material with units (W/g magnetic material). When considering ferrofluids, the thermal loss power is often normalized by iron mass. For the MnFe_2_O_4_ nanoparticles the measured iron mass (11.25 g) was used to calculate total mass of magnetic material using the stoichiometric Mn to Fe ratio (1:2), yielding ~16.8 g magnetic material in the MnFe_2_O_4_ nanoparticle sample.

**Figure 7:**
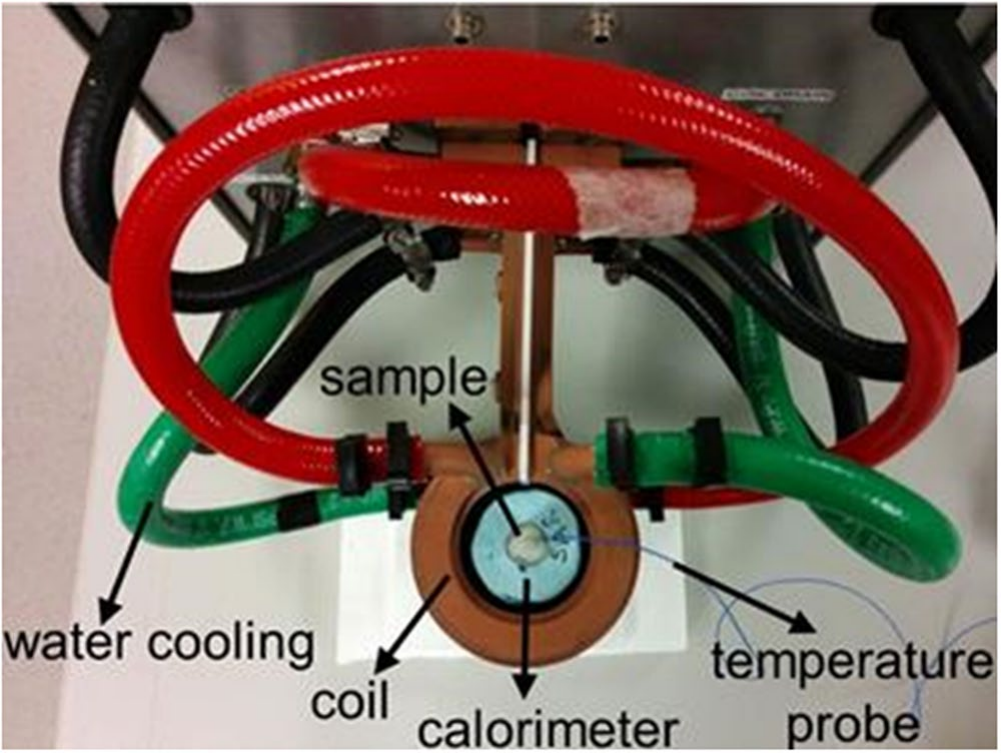
Experimental setup for calorimetric SLP measurements.

To capture heat generated by the sample and to reduce temperature changes inside the sample due to coil temperature fluctuations, an insulating sample holder was placed inside the vertical coil. A standard 12 mm (5 ml) polystyrene tube, containing exactly 1 g of a particular magnetic nanoparticle solution, was inserted in the center of the sample holder. A fiber optic temperature probe (FISO Technologies, Quebec City, Canada) was immersed in the magnetic nanoparticle solution. The temperature probe was routinely calibrated and was used for every SLP measurement. A parafilm seal fixed the position of the fiber optic temperature probe inside the solution, minimizing systemic errors. The experimental setup is depicted in Fig. 7.

Individual measurements consisted of different steps. First, the temperature of the magnetic nanoparticle was monitored before heating to ensure it was constant, i.e. maximum deviation of 0.02 °C in 30 s, indicating that the system was equilibrated with the environment. Subsequently, the alternating magnetic field was applied by powering up the supply until the temperature in the sample reached 45 °C or until 100 s elapsed. Sample temperature was recorded at 0.4-s intervals. This process was repeated for each sample and at each frequency and magnetic field amplitude combination.

### SLP estimation and analysis

Calorimetric SLP measurements are based on the expression $SLP=\frac{C}{{m}_{Fe}}\frac{{\rm{\Delta }}T}{{\rm{\Delta }}t}$, which is only valid in (quasi-)adiabatic regime (*T* = temperature, *t* = time, *C* = sample heat capacity, *m*_*Fe*_ = equivalent iron mass of sample). The temperature increases of the nanoparticle sample (*Δ* *T*_*MNP*_) and the associated water sample (*Δ* *T*_*w*_) (at the same frequency and amplitude) were obtained by subtracting the initial temperatures from their measured temperatures at each time, *t*, Δ*T*_*MNP/w*_(*t*) = *T*_*MNP/w*_(*t*) − *T*_*MNP/w*_(0). Subsequently, the temperature increase of the water blank was subtracted from that of the nanoparticle sample, resulting in the net temperature increase, *Δ* *T*_*net*_(*t*) = *Δ* *T*_*MNP*_(*t*) − *Δ* *T*_*w*_(*t*). The SLP was estimated from the slope of this net temperature increase. First, we performed a linear-least-squares fit of the net temperature increase to determine the slope in a chosen time range (from *t*_*start*_ to *t*_*end*_) (Fig. 1a). To identify whether the chosen time range met (quasi)-adiabatic conditions, we inspected the first derivative of the net temperature increase. Due to the discrete nature of the temperature measurements (temporal resolution of 0.4 s), the first derivative can be approximated as the incremental net temperature increase per time step, i.e. the ratio of the difference between the net temperature increase at time *t*_*n+1*_ and the net temperature increase at the previous time step *t*_*n*_ and the time difference itself, [*Δ* *T*_*net*_(*t*_*n+1*_) − *Δ* *T*_*net*_(*t*_*n*_)]/(*t*_*n+1*_ − *t*_*n*_). We determined both the mean value of the rate of rise, and the y-intercept of a linear least square fit of the first derivative of the net temperature increase in the same time range (from *t*_*start*_ to *t*_*end*_) (Fig. 1b). The heating in the chosen time range was considered to be (quasi)-adiabatic when these values were within 5% of the net temperature increase slope. Subsequently, the net temperature rise slope in the (quasi-)adiabatic time region was used to calculate the SLP, with the heat capacity of the sample, *C*, assumed to be that of an equivalent amount of water (*C* = *c*_*water*_ * *m*_*sample*_, specific heat capacity of water at 20 °C and 1013.25 hPa = 4.18 J/g/°C, was used) and the iron mass based on the iron concentration of Table 1.

### Computational methods and MATLAB code

The procedure to determine ranges of values used for estimating SLP was automated in MATLAB (R2015b; Mathworks, Natick, MA, USA) and repeated for every possible time range, i.e. for every combination of *t*_*start*_ and *t*_*end*_. The minimum time difference between *t*_*end*_ and *t*_*start*_ was set to 6 s, whereas the maximum time difference was 100 s, the maximum duration of the heating experiment. Every time range that met our criterion for (quasi)-adiabatic conditions independently resulted in a new SLP-value.

### Statistical methods and analysis

The average of all obtained values satisfying the (quasi)-adiabatic criteria was defined as the measured SLP-value. The standard deviation of these values was used to generate error bars in the figures.

To further demonstrate the inherent variation in SLP values for a specific magnetic nanoparticle sample exposed to a magnetic field with a specific amplitude and frequency, we displayed all obtained values using the “box-and-whisker plot” representation. In this plot, the blue horizontal lines at the bottom and at the top of the box are always the first and third quartiles (SLP_25%_ & SLP_75%_), respectively, whereas the median (SLP_50%_) is depicted by the red horizontal line within the box. The average SLP value is denoted by a green dot inside the box. The difference between the end of the whiskers (adjacent values) and the box edges can maximally be 1.5 times the interquartile distance, i.e. SLP_75%_ - SLP_25%_. Any SLP value that was not included between the whisker ends was considered to be an outlier (red plus-sign).

Another representation to illustrate variance is the histogram plot. The histogram graphically represents (see Supplementary Materials Figures S2) the distribution of SLP values (relative frequency). Besides the histogram, an additional probability density estimate was provided based on a normal (Gaussian) kernel function (red curve of Figures S2). The average SLP was shown as a green vertical line in the histogram plots.

## Electronic supplementary material

Supplementary Data for Experimental estimation and analysis of variance of the measured loss power of magnetic nanoparticles
