# Supplementary material for: Experimental estimation and analysis of variance of the measured loss power of magnetic nanoparticles
Source: Sci Rep. 2017 Jul 27;7:6661. doi: 10.1038/s41598-017-07088-w (PMC5532265; doi:10.1038/s41598-017-07088-w)
Supplement: Supplementary file 1 — Supplementary Data for Experimental estimation and analysis of variance of the measured loss power of magnetic nanoparticles [file 41598_2017_7088_MOESM1_ESM.pdf]

Supplementary Data for

Experimental estimation and analysis of variance of the measured loss power of magnetic nanoparticles

Frederik Soetaert, Sri Kamal Kandala, Andris Bakuzis, Robert Ivkov

The inherent variance of data can also be displayed with histogram plots. The number of bins, and thus the bin width, is determined using Sturges' formula: $k = 1 + \lceil \log_2 n \rceil$, where *n* equals the amount of possible SLP values. **Fig. S2 a** depicts the relative frequency of the SLP values meeting the (quasi)-adiabatic criterion of the same BNF-Dextran heating experiment as in **Fig. R2 a** (150 kHz and 20 kA/m). These data can be viewed as representative of an estimation of the probability density of the SLP distribution. The red line is a probability density estimate based on a normal kernel function and is displayed here to demonstrate the trend. At this frequency and amplitude, the SLP distribution is tri-modal. The average SLP value (vertical green line in **Fig. S2 a-d**) is situated between two major peaks with a smaller third peak at lower SLP values, corresponding to the trailing outliers in **Fig. R2 a**. Analogously, the histogram plot of JHU nanoparticles (**Fig. S2 b**) shows that the SLP distribution is bi-modal, with a few trailing outliers at lower SLP values. The histogram plot of nanomag®-D-spio's (**Fig. S2 c**) highlights the large variance of the SLP measurements , as the probability density estimate is not smooth. Outliers are indeed scattered all over the place in **Fig. R2 c**. In the case of $MnFe_2O_4$ nanoparticles (**Fig. S2 d**), the histogram plot indicates a bi-modal SLP distribution with two main peak around the average SLP value, with no trailing outliers (**Fig. R2 d)**.

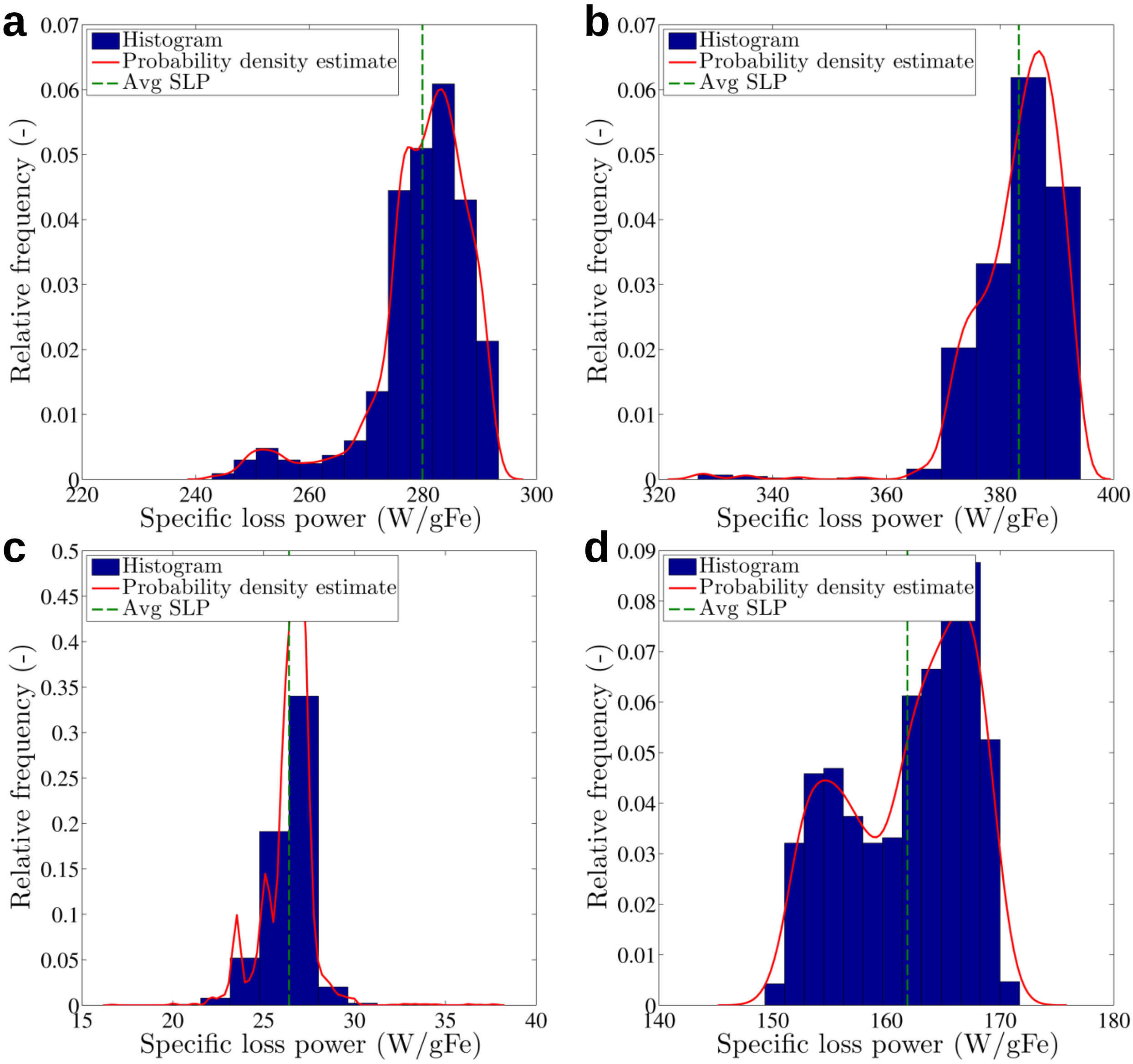

Figure S1: Histogram plot of the relative frequency of SLP values that meet the criterion for the heating experiment of BNF-Dextran (a), JHU (b), nanomag®-D-spio (c), and $MnFe_2O_4$ nanoparticles (d) at 150 kHz and 20 kA/m. The red curve is a probability density estimate based on a normal kernel function. The green vertical line indicates the average SLP value which is considered to be the measured SLP value. This histogram indicates the large amount of possible SLP values one can deduce from a single experiment, therefore necessitating the calculation of the SLP in every (quasi)-adiabatic time range.

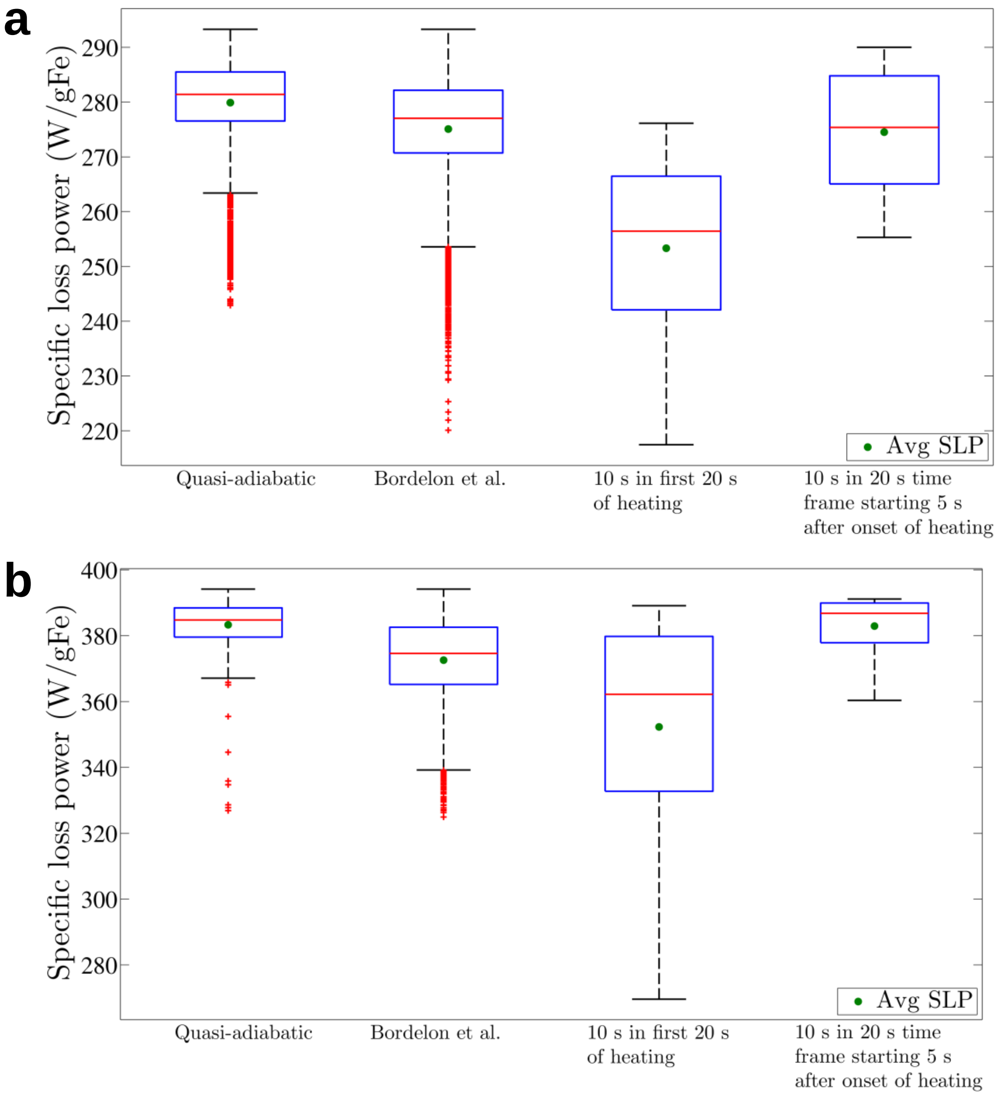

a
Specific loss power (W/gFe)
290
280
270
260
250
240
230
220
Avg SLP
Quasi-adiabatic
Bordelon et al.
10 s in first 20 s of heating
10 s in 20 s time frame starting 5 s after onset of heating
b
Specific loss power (W/gFe)
400
380
360
340
320
300
280
Avg SLP
Quasi-adiabatic
Bordelon et al.
10 s in first 20 s of heating
10 s in 20 s time frame starting 5 s after onset of heating

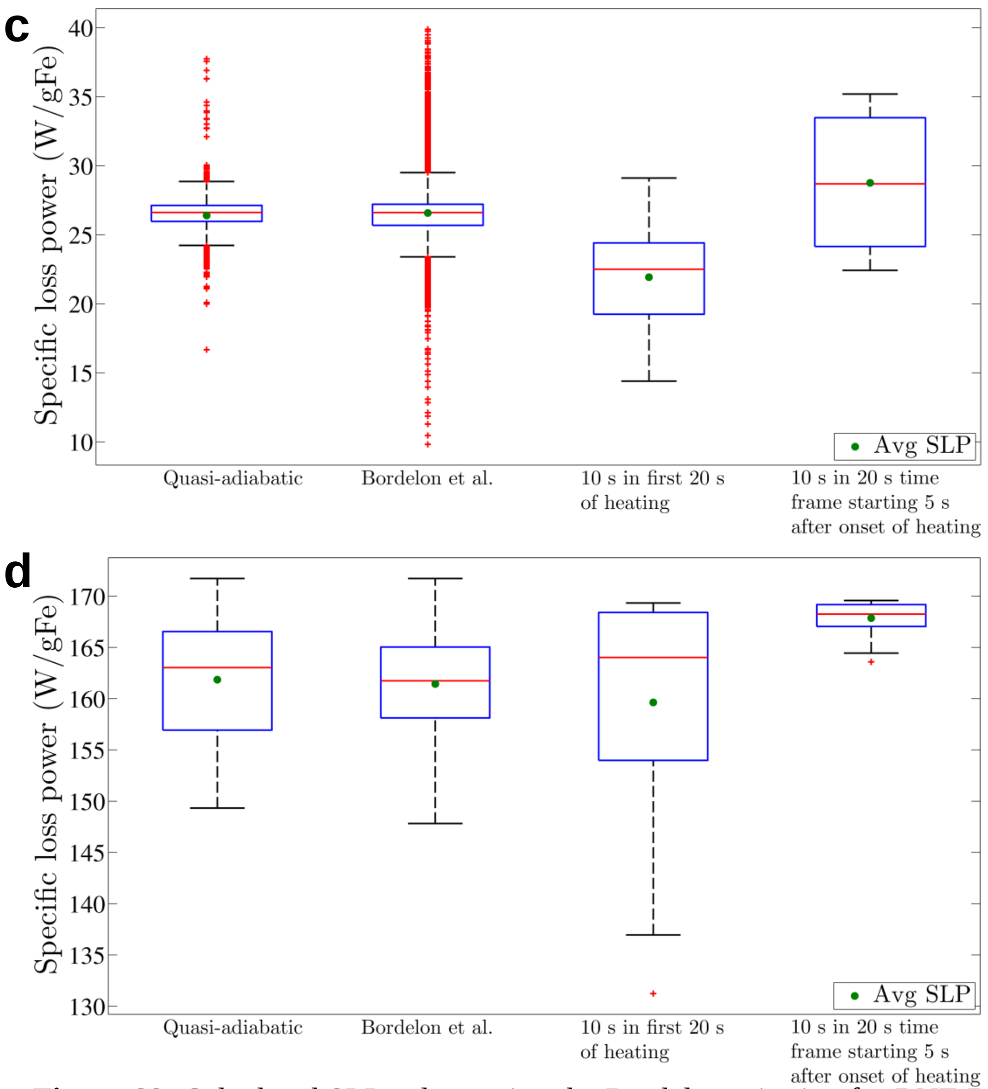

Figure S2: Calculated SLP values using the Bordelon criterion for BNF-Dextran (a), JHU (b), nanomag®-D-spio (c), and $MnFe_2O_4$ nanoparticles (d) at 150 kHz and 20 kA/m. The (quasi)-adiabatic criterion used in the current study is more stringent than that used by Bordelon, *et al.*[12], leading to a slight underestimation of the SLP, compared to results obtained using the current quasi-adiabatic criterion. The third criterion represents all 10 s periods in the first 20 s of heating, whereas the fourth criterion also includes every 10 s period, but in a 20 s timeframe starting 5 s after onset of heating.

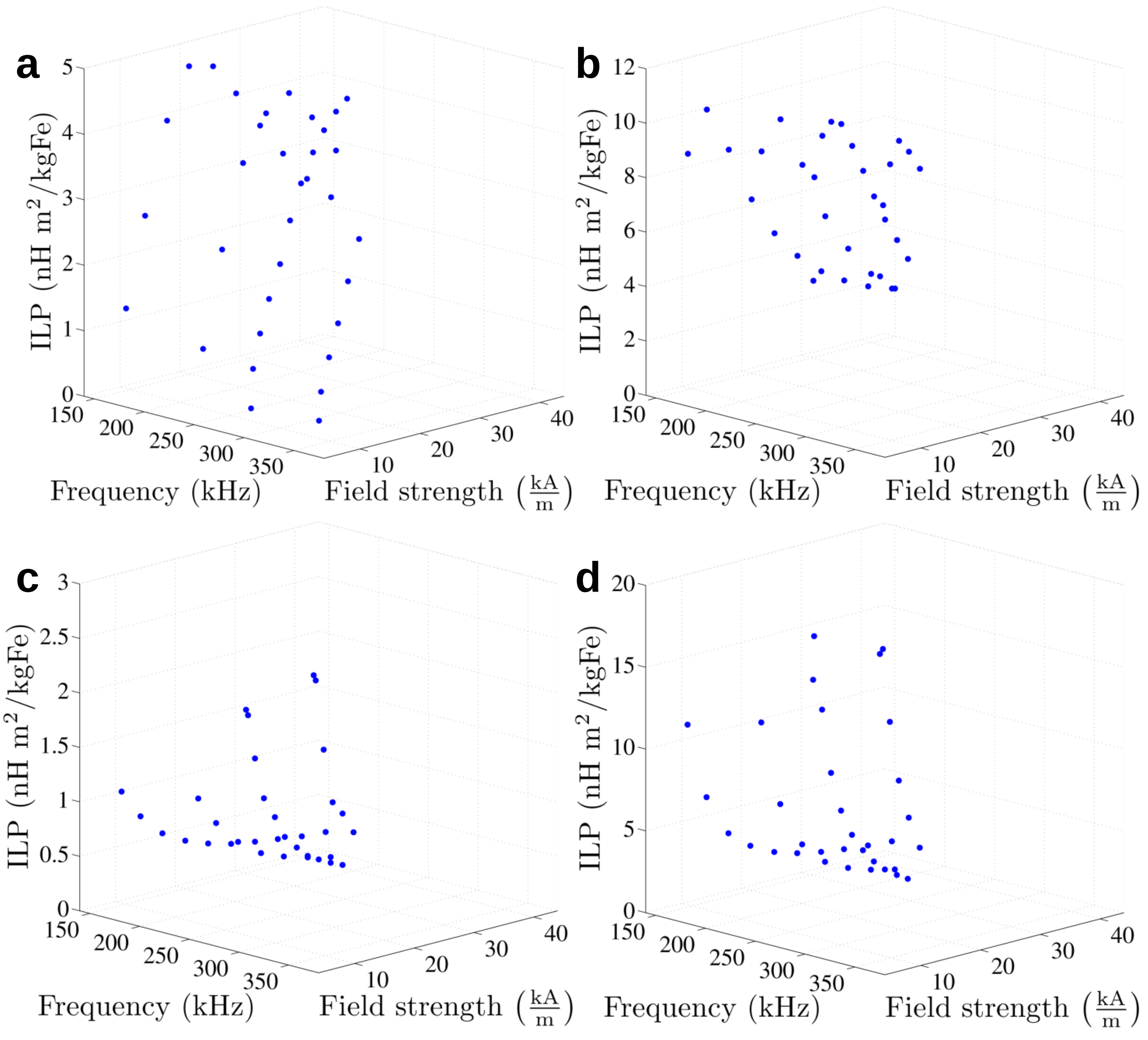

Figure S3: Intrinsic loss power values calculated based on experimentally determined specific loss power values in a range of frequencies f and field strengths H (a: BNF Dextran, b: JHU, c: nanomag®-D-spio, d: $MnFe_2O_4$ nanoparticles). The calculated ILP values (blue dots) do not appear to be coplanar for each individual magnetic nanoparticle.
